# Selinexor and COVID-19: The Neglected Warden

**DOI:** 10.3389/fphar.2022.884228

**Published:** 2022-04-26

**Authors:** Gomaa Mostafa-Hedeab, Hayder M. Al-kuraishy, Ali I. Al-Gareeb, Nermeen N. Welson, Gaber El-Saber Batiha, Carlos Adam Conte-Junior

**Affiliations:** ^1^ Pharmacology Department & Health Research Unit, Medical College, Jouf University, Jouf, Saudi Arabia; ^2^ Pharmacology Department, Faculty of Medicine, Beni-Suef University, Beni Suef, Egypt; ^3^ Department of Clinical Pharmacology and Medicine, College of Medicine, ALmustansiriyia University, Baghdad, Iraq; ^4^ Department of Forensic Medicine and Clinical Toxicology, Faculty of Medicine, Beni-Suef University, Beni Suef, Egypt; ^5^ Department of Pharmacology and Therapeutics, Faculty of Veterinary Medicine, Damanhour University, Damanhour, Egypt; ^6^ Center for Food Analysis (NAL), Technological Development Support Laboratory (LADETEC), Federal University of Rio de Janeiro (UFRJ), Cidade Universitária, Rio de Janeiro, Brazil

**Keywords:** SARS-CoV-2, COVID-19, Selinexor, nuclear exportin-1, inflammation

## Abstract

A novel severe acute respiratory distress syndrome coronavirus type 2 (SARS-CoV-2) has been confirmed as the cause of the global pandemic coronavirus disease 2019 (COVID-19). Different repurposed drugs have been trialed and used in the management of COVID-19. One of these agents was the anti-cancer Selinexor (SXR). SXR is an anti-cancer drug that acts by inhibition of nuclear exportin-1 (XPO1), which inhibits transport of nuclear proteins from the nucleus to the cytoplasm, leading to the induction of cell-cycle arrest and apoptosis. XPO1 inhibitors had antiviral effects, mainly against respiratory syncytial virus (RSV) and influenza virus. SXR inhibits transport of SARS-CoV-2 nuclear proteins to the cytoplasm with further inhibition of SARS-CoV-2 proliferation. SXR has the ability to prevent the development of a cytokine storm in COVID-19 by inhibiting the release of pro-inflammatory cytokines with the augmentation release of anti-inflammatory cytokines. In conclusion, SARS-CoV-2 infection is linked with activation of XPO1, leading to the triggering of inflammatory reactions and oxidative stress. Inhibition of XPO1 by Selinexor (SXR), a selective inhibitor of nuclear export (SINE), can reduce the proliferation of SARS-CoV-2 and associated inflammatory disorders. Preclinical and clinical studies are warranted in this regard.

## Introduction

A novel severe acute respiratory syndrome coronavirus type 2 (SARS-CoV-2) has been confirmed as the cause of a global pandemic coronavirus disease 2019 (COVID-19) (Al-Kuraishy et al., 2021a), a primary respiratory disease that causes viral pneumonia and leads to acute lung injury (ALI) and acute respiratory distress syndrome (ARDS) in severe cases (Al-Kuraishy et al., 2021b). However, extra-pulmonary manifestations of COVID-19 have been shown to include neurological, cardiovascular, gastrointestinal, renal, and metabolic complications (Al-Kuraishy et al., 2021c). Of note, COVID-19 may be associated with several complications, including cytokine storm and multi-organ failure (MOF) (Al-Kuraishy et al., 2021f). Severe COVID-19 syndromes can result in acute kidney injury (Al-Kuraishy et al., 2021g; Al-Kuraishy et al., 2021h), coagulopathy and ischemic stroke (Al-Kuraishy et al., 2021j), endocrine dysfunction (Al-Kuraishy et al., 2021k), sympathetic storm (Al-Kuraishy et al., 2021L), and dysautonomia (Al-Kuraishy et al., 2021L). These complications arise as a result of the direct SARS-CoV-2 cytotopathic effect as well as the associated hyperinflammation and cytokine storm (Al-Kuraishy et al., 2021L).

The clinical presentation of COVID-19 is an asymptomatic or mild form of flu-like illness in the majority of cases (85%) like headache, anosmia, fever, dry cough, myalgia, joint pain, and sweating (Parasher, 2021). However, approximately 15% of COVID-19 patients may experience a severe form due to the development of ALI, leading to severe dyspnea and hypxemia that requires hospitalization (Mehta et al., 2021). Notably, 3–5% of severe COVID-19 patients may progress to a critical stage that requires mechanical ventilation and intensive care admission due to the progression of ARDS (Schönfeld et al., 2021).

Different biomarkers, including D-dimer, lactate dehydrogenase (LDH), C-reactive protein (CRP), serum ferritin, and procalcitonin levels, are elevated in COVID-19 patients and are associated with disease severity and clinical outcomes. For example, LDH reflects the severity of ALI and D-dimer reflects the underlying coagulopathy (Copaescu et al., 2021).

The pathogenesis of SARS-CoV-2 infection occurs by the binding of this virus to the angiotensin converting enzyme 2 (ACE2), which is highly expressed in various cell types, including immune cells (Woodby et al., 2021). SARS-CoV-2 infection causes immunological and inflammatory responses that resolve after viral clearance (Woodby et al., 2021). However, in some cases, an exaggerated immune response and the release of large amounts of pro-inflammatory cytokines may occur, resulting in hyperinflammation and cytokine storm (Lin et al., 2021). Moreover, severe SARS-CoV-2 infection may induce the development of oxidative stress by inducing the generation of reactive oxygen species (ROS) and the reduction of human body antioxidant capacity (Al-Kuraishy et al., 2022n). In severe SARS-CoV-2 infection, oxidative stress increases COVID-19 severity by triggering the release of pro-inflammatory cytokines and the spread of endothelial dysfunction and pulmonary microthrombosis (Fodor et al., 2021). Therefore, direct and indirect effects of SARS-CoV-2 infection may lead to systemic and oxidative stress effects with the development of MOF (Lin et al., 2021).

Thus, direct anti-SARS-CoV-2 and anti-inflammatory agents can decrease the pathogenic effect of SARS-CoV-2 infection and associated inflammatory complications. Different repurposed drugs like chloroquine, hydroxychloroquine, remdesivir, silvestrol, saracatinib, favipiravir, and azithromycin have been trialed and used in the management of COVID-19 (De et al., 2021). By virtual screening of 6,218 repurposed and clinical trial drugs for COVID-19, tipifarnid, omipalisib, and emodin had anti-SARS-CoV-2 activities (Jang et al. 2021). The activity of omipalisib is more potent by about 200 times than remdesivir against SARS-CoV-2 in human lung cells (Jang et al. 2021). These repurposed trailed drugs show potent synergistic effects with remdesivir against SARS-CoV-2 (Jang et al. 2021).

One of these agents was the anti-cancer selinexor (SXR), which was recently approved by the Food and Drug Administration (FDA) in 2019 for the treatment of refractory multiple myeloma (MM) (Syed, 2019).

Therefore, the objective of the present report was to illustrate the potential role of SXR in the pathogenesis of SARS-CoV-2 infection.

### Pharmacology of Selinexor

**FIGURE 1:**
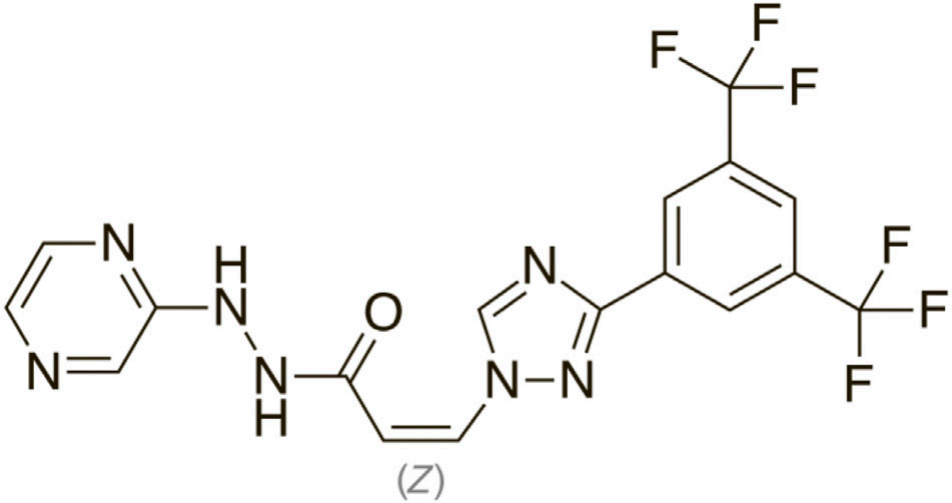
Chemical structure of Selinexor.

The chemical formula for selinexor (SXR) is [Z-3 (3-(3, 5-bis (trifluromethyl-1, 2, 4-triazol-1-yl)-N-pyrazin-2-ylprop-2-enehydrazide] (Figure 1) (Chari et al., 2019).

**FIGURE 2:**
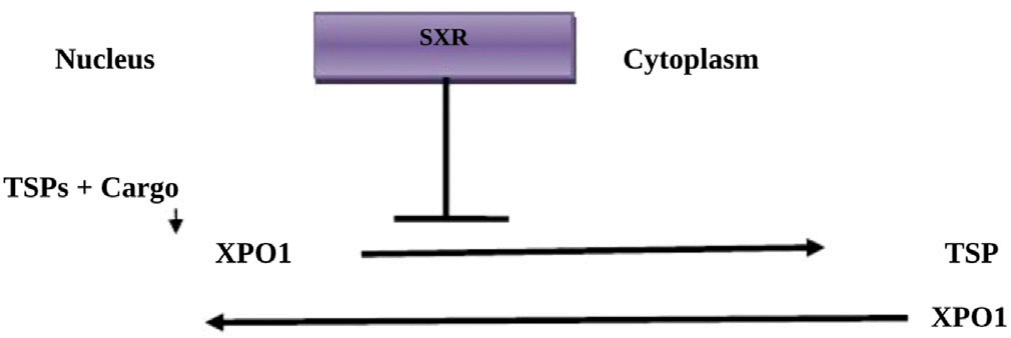
Mechanism of Selinexor action: Tumor suppressor proteins (TSPs) and cargo proteins bind to exportin-1 (XPO1), which transports them to the cytoplasm. Selinexor (SXR) inhibits XPO1, preventing the transport of TSPs.

SXR is an anti-cancer drug that acts by inhibition of nuclear exportin-1 (Chari et al., 2019). SXR is regarded as a selective inhibitor of nuclear export known as exportin-1 (XPO1), which inhibits transport of nuclear proteins from the nucleus to the cytoplasm, leading to the induction of cell-cycle arrest and apoptosis (Chari et al., 2019). SXR is also known as chromosome region maintenance 1 protein (CRM1) and inhibits different types of tumor suppressor proteins (TSPs), including p21, p53, pRB, FOXO, and BRCA1/2, restoring the process of endogenous tumor suppression (Figure 2) (Podar et al., 2020).

**FIGURE 3:**
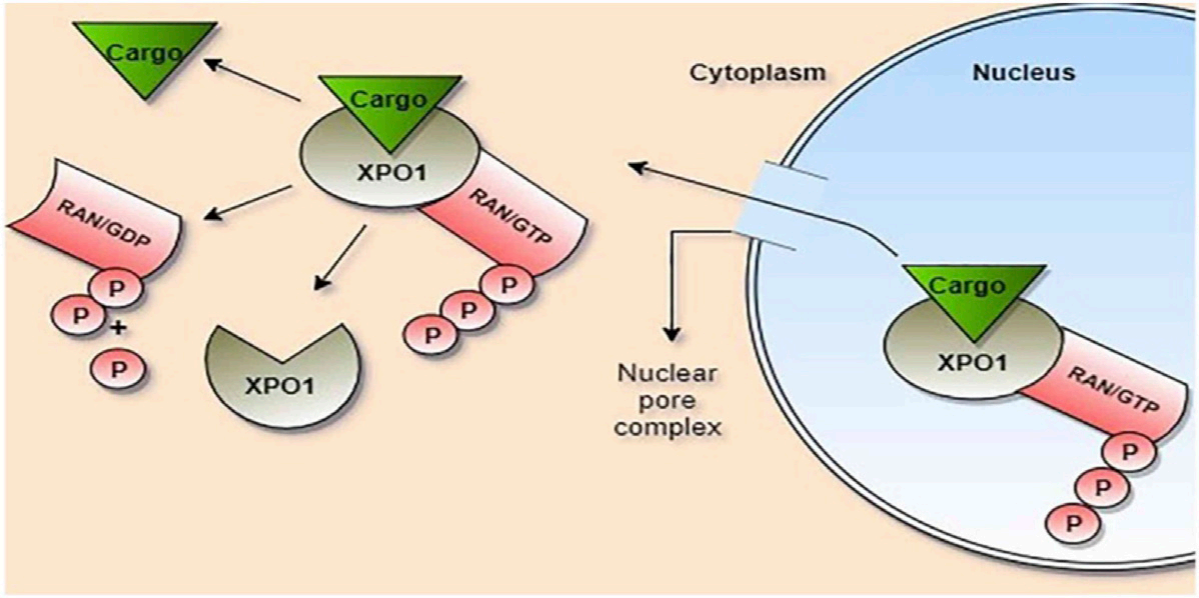
Role of exportin-1 (XPO1) in the transport of cargo proteins: Cargo, ras-related nuclear protein (Ran), and guanosin triphosphate (GTP) bind to XPO1, forming a large complex which passes through the nuclear pore complex. Ran/GTP is hydrolyzed to Ran/GDP within the cytoplasm with the release of cargo and phosphate (P).

SINE is a class of specific small molecules that have anti-inflammatory and antiviral properties (Widman et al., 2018). XPO1 induces accumulation of nuclear proteins within the nucleus of cancer cells with increasing expression of tumor suppressor genes (Podar et al., 2020). XPO1 mediates transport and export of leucine-rich nuclear export signals (NES) containing proteins and RNA transcripts (Widman et al., 2018). Of interest, nucleocytoplasmic transport occurs *via* a specific system called the nuclear pore complex, which permits passive diffusion of small molecules and active transport of large cargo proteins (Figure 3). As well, the nucleocytoplasmic transport trafficking pathway is involved in the propagation of the inflammatory process and the pathogenesis of viral infections (Widman et al., 2018). Mathew et al.'s study confirmed that XPO1 is necessary for the viral life cycle Mathew and Ghildyal (2017).

SXR was the first selective inhibitor of nuclear export (SINE) indicated for the treatment of refractory multiple myeloma (MM) in patients who have not responded to proteasome inhibitors and immunotherapy (Chari et al., 2019). SXR is also indicated in the management of refractory and relapsing B-cell lymphoma (Kalakonda et al., 2020). Under the name of KPT-330, SXR was tested in different animal model studies in the treatment of solid tumors and chronic leukemia (Parikh et al., 2014). Moreover, different preclinical and ongoing clinical studies have shown the effectiveness of SXR and other SINEs in the treatment of various malignancies. For example, SXR in a phase II study was effective against different gynecological tumors like cervical, ovarian, and endometrial cancers (Vergote et al., 2020). As well, SXR has been shown to be of interest in treating acute myeloid leukemia (Talati and sweet 2018).

SXR is given orally at a dose of 80 mg once weekly. Its absorption is not affected by food, and it has a 95% protein binding capacity with a 125-L volume of distribution. SXR has a half-life of 6–8 h and is mostly eliminated by bile after being metabolized by hepatic glucoronosyl transferase (Bader et al., 2021). SXR is associated with some adverse effects, including nausea, vomiting, leucopenia, thrombocytopenia, anemia, and hyponatremia (Bader et al., 2021). SXR is associated with some adverse effects, including nausea, vomiting, leucopenia, thrombocytopenia, anemia, and hyponatremia (Bader et al., 2021). In a BOSTON clinical study used to evaluate FDA-approved drugs in patients with MM following at least one prior treatment with SXR in combination with dexamethasone and bortezomib, it was revealed that the most common adverse effects were gastrointestinal disorders and cytopenia (Grosicki et al., 2020). Besides, other adverse effects have been reported in this study, like fatigue (59%), nausea (50%), thrombocytopenia (43%), lymphopenia (38%), anorexia (35%), and diarrhea (32%) (Grosicki et al., 2020). Furthermore, serious adverse effects have been reported in 46% of patients treated by SXR, including gastrointestinal toxicity, neurological toxicity, and secondary infections (Grosicki et al., 2020).

Regarding drug interactions of SXR, antifungal drugs like posaconazole, itracanazole, and isavuconazole increase the plasma concentration of SXR by inhibiting the CYP3A4 enzyme, which is involved in the metabolism of SXR (Zhou et al., 2021). Bader et al., 2021 recently found that SXR pharmacokinetics are less affected by other drugs and organ dysfunctions. (Bader et al., 2021).

### Antiviral and Anti-Inflammatory Effects of Selinexor

It has been reported that XPO1 inhibitors have antiviral effects mainly against respiratory syncytial viruses (RSV) and influenza viruses (Jorquera et al., 2019; Uddin et al., 2020). An *in vitro* study illustrated that KPT-335, a SINE, prevents transport of RSV M protein from the nucleus to the cytoplasm (Jorquera et al., 2019). Watanabe *et al.* found that leptomycin B, a SINE, attenuates influenza virus infection through inhibition of viral ribonucleoprotein export from the nucleus to the cytoplasm (Watanabe et al., 2001). XPO1 cargo proteins are essential for the regulation of vial proliferation and maturation (Uddin et al., 2020).

Notably, coronavirus infection stimulates translocation of different cargo proteins through an XPO1-dependent pathway (Uddin et al., 2020). As well, XPO1 inhibitors attenuate replication of human immunodeficiency virus type 1 (HIV-1) through inhibition of nuclear export of HIV intron-containing RNA (Boons et al., 2015). Indeed, nucleo-cytoplasmic transport blockers such as leptomycin B and ivermectin reduce the replication of equine herpes virus type 1 (Slonska et al., 2013).

It has been reported that verdinexor and other SINE agents were effective against *in vitro* replication and pathology of influenza virus infection (Perwitasari et al., 2016). Similarly, an *in vivo* study demonstrated that verdinexor can reduce viral burden and virus-induced lung inflammation with a subsequent decreased mortality rate even when given 4 days following influenza virus infection (Perwitasari et al., 2016). Verdinexor and SXR are closely related compounds; they have similar pharmacokinetic and pharmacodynamic properties (Perwitasari et al., 2016).

Different viruses, including SARS-CoV-2, require nuclear XPO1 to carry their proteins. For example, envelop and nucleoproteins need XPO1 for proper action (Zhou et al., 2020). Jorquera *et al.* found that inhibition of XPO1 by leptomycin B and other natural products results in apoptosis of SARS-CoV infected cells Jorquera et al. (2019). SXR has the ability to inhibit SARS-CoV-2 replication by inhibiting the release of pro-inflammatory cytokines and activating the release of anti-inflammatory cytokines (de Lemos et al., 2003).

SXR, on the other hand, has potent anti-inflammatory effects by inhibiting nuclear factor B (NF-B), resulting in the inhibition of IL-1, IL-6, and interferon gamma (INF-γ) release (Kashyap et al., 2016). Besides, SXR has an antioxidant effect by inducing the release of nuclear erythroid factor 2 (Nrf2) (Tajiri et al., 2016). Also, SXR has anti-inflammatory and cytoprotective effects by activating peroxisome proliferator activator receptor gamma (PPAR-γ) (Umemoto and Fujiki, 2012). Wu *et al.* revealed that SXR had the ability to attenuate lipopolysaccharide (LPS)-induced peritoneal sepsis in mice Wu et al. (2018).

These observations suggest that SXR has antiviral and anti-inflammatory effects, thereby enabling it to mitigate different viral infections and associated inflammatory disorders.

### Role of Selinexor in COVID-19

SXR covalently binds the cysteine 528 residue of XPO1’s cargo binding pocket, inhibiting nuclear protein transport, including SARS-CoV-2 viral protein, nucleocapsid proteins, and ORF3b/ORF9b, which block host immune response (Kashyap et al., 2021). Uddin et al. confirmed the dose-dependent manner of SXR in the inhibition of the SARS-CoV-2 proliferation (Uddin et al., 2020).

Definitely, XPO1 inhibitors can limit the interaction between the SARS-CoV-2 viral protein and host cell receptors (Gordon et al., 2020). At present, ACE2 is regarded as a cargo protein transported by XPO1 to express on the cell membrane (Kashyap et al., 2021). Therefore, inhibition of nuclear XPO1 by SXR may limit expression of ACE2 and its interaction with SARS-CoV-2 (Kashyap et al., 2021). However, this effect was transient and not completely blocked by SXR therapy.

Moreover, a recent study confirmed that neuropilin-1 (NRP-1) may act as a receptor and facilitate entry of SARS-CoV-2 into the host cell *via* the interaction between its B1 domain and the S1 subunit of the SARS-CoV-2 spike glycoprotein. Thus, NRP-1 could be a potential target for SARS-CoV-2 infection. It has been shown by *in vivo* and *in vitro* testing that SXR has the ability to block the NRP-1 receptor (Charoute et al., 2022). Therefore, SXR can reduce the pathogenesis of SARS-CoV-2 infection independent of the ACE2 pathway. However, the effect of SXR on other proposed SARS-CoV-2 receptors has been suggested but not documented (Charoute et al., 2022).

Of note, XPO1 inhibitors block the replication of SARS-CoV by inhibiting the export of viral nuclear proteins ORF3, N, 9B, and S proteins (Agree, 2020). Lee and others revealed that SARS-CoV-2 ORF6, which uses XPO1, is the most cytotoxic protein in human 293-T cells, and use of SXR may attenuate this *in vitro* toxicity (Lee et al., 2021). Therefore, SXR could be the potential therapy to prevent the SARS-CoV-2-induced cytopathic effect. SARS-CoV ORF proteins, in particular, have been linked to significant cytotoxicity (Khan et al., 2006). To evade host immune response and suppress immune response to the invading virus, SARS-CoV-2 ORF6 antagonizes INF signaling and interacts with the nuclear pore protein NUP98-RAE1 (Miorin et al., 2020). Therefore, prevention of the entrance of SARS-CoV-2 via importin inhibitors like ivermectin or using XPO1 inhibitors like SXR could be effective against SARS-CoV-2 infection (Al-Kuraishy et al., 2020 d).

Moreover, SXR has the ability to prevent the development of a cytokine storm in COVID-19 by inhibiting the release of pro-inflammatory cytokines since SINE had the ability to inhibit the release of inflammatory cytokines in different experimental studies (Perwitasari et al., 2016; Jorquera et al., 2019). Kashyap *et al.* experimental study demonstrated that SXR can inhibit SARS-CoV-2 proliferation and attenuate associated inflammation by inhibiting pro-inflammatory cytokine release and augmenting anti-inflammatory cytokine release Kashyap et al. (202).

Regarding the role of nuclear proteins in SARS-CoV-2 infection, it has been shown that p53 inhibits expression of ACE2 and interaction with SARS-CoV-2 (Zhang et al., 2021). ACE2 seems to be protective rather than harmful by attenuating inflammatory and coagulation disorders in COVID-19 by metabolizing vasoconstrictor angiotensin II (AngII) to anti-inflammatory Ang1-7 (Al-Kuraishy et al., 2020 e). Subhash *et al.* revealed that SXR and other SINE can inhibit transport of p53 in gastric carcinoma, resulting in nuclear accumulation of p53 with induction of apoptosis and cell-cycle arrest Subhash et al. 2(2018). SARS-CoV-2 and other coronaviruses induce cell-cycle arrest and apoptosis through the induction of p53 (Hemmat et al., 2021). Therefore, inhibition of the p53 pathway by SXR may decrease SARS-CoV-2-induced apoptosis in COVID-19.

Notably, SXR can reduce oxidative and inflammatory disorders in COVID-19 by activating Nrf2 and PPAR-γ (Umemoto and Fujiki, 2012; Tajiri et al., 2016). Several studies (Filgueira et al., 2021; Pivonello et al., 2021) found that high inflammatory and oxidative burdens in SARS-CoV-2 infection were associated with COVID-19 severity.

**FIGURE 4:**
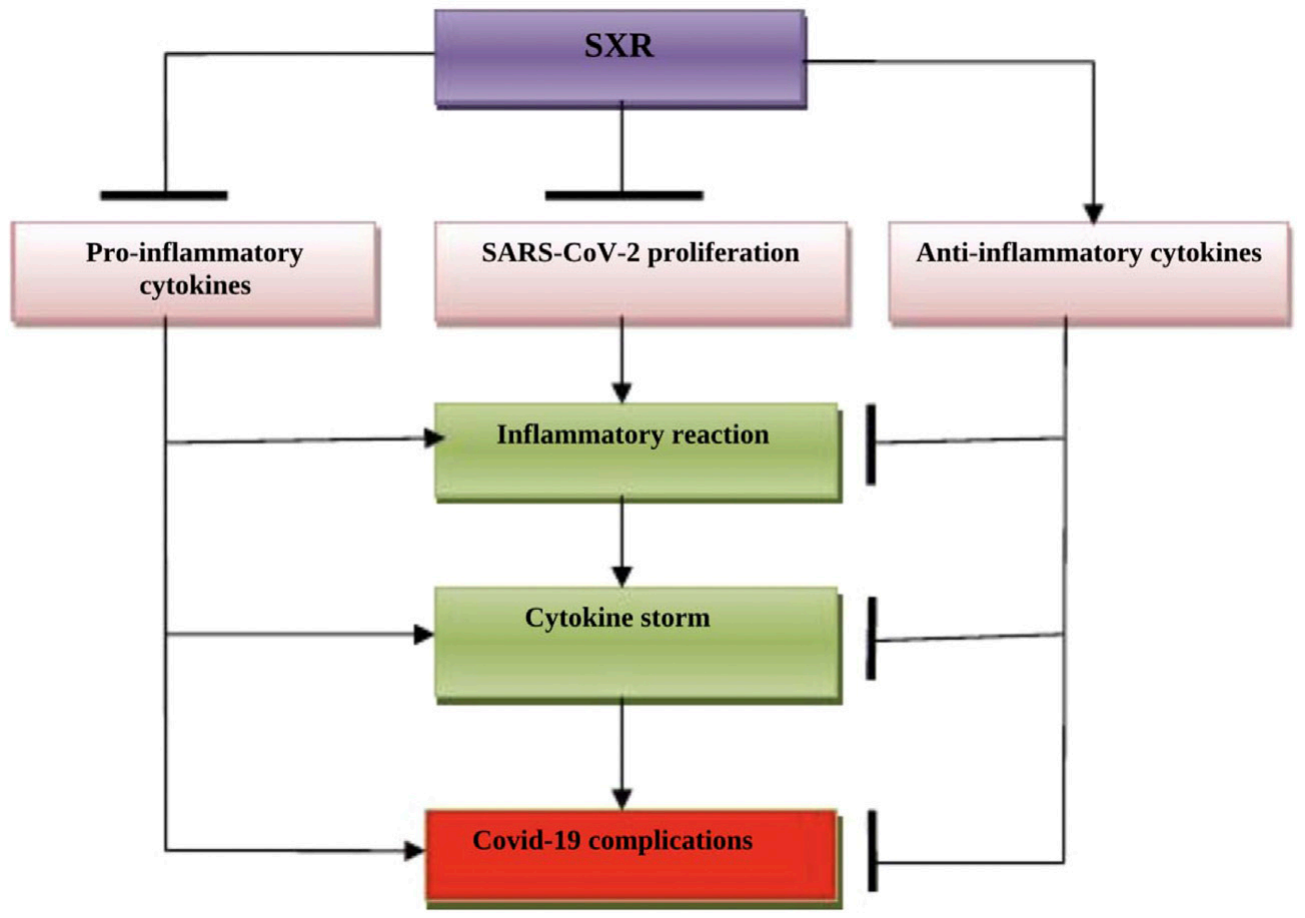
The possible role of Selinexor (SXR) in COVID-19: SXR inhibits the proliferation of SARS-CoV-2 and the release of pro-inflammatory cytokines. SXR activates the release of anti-inflammatory cytokines, thereby preventing the development of a cytokine storm and COVID-19 severity.

Taken together, SXR could be a potential candidate in the management of COVID-19 because of its anti-inflammatory, antioxidant, and anti-SARS-CoV-2 effects (Figure 4).

The present review has several limitations, including a paucity of clinical studies and long-term adverse effects of SXR were not evaluated. Despite these limitations, this review highlighted the potential role of SXR in the management of COVID-19 and could be a preliminary report evoking researchers for large-scale prospective studies.

## Conclusion

SARS-CoV-2 infection is linked with activation of XPO1, leading to the triggering of inflammatory reactions and oxidative stress. Inhibition of XPO1 by selinexor (SXR), a selective inhibitor of nuclear export (SINE), can reduce the proliferation of SARS-CoV-2 and associated inflammatory disorders. Preclinical and clinical studies are warranted in this regard (Al-Kuraishy et al., 2021m).
